# Supplementary material for: Last Resort from Nursing Shortage? Comparative Cost Analysis of Open vs. Robot-Assisted Partial Nephrectomies with a Focus on the Costs of Nursing Care
Source: Cancers (Basel). 2023 Apr 14;15(8):2291. doi: 10.3390/cancers15082291 (PMC10137045; doi:10.3390/cancers15082291)
Supplement: Supplementary file 1 [file cancers-15-02291-s001.zip › cancers-2337796-supplementary.pdf]

Last resort from nursing shortage? Comparative cost analysis of open vs. robot-assisted partial nephrectomies with a focus on the cost of nursing care

Table S1. Overview of the intensive care and performance recording system (Intensivpflege- und Leistungserfassungssystem, INPULS®) and the respective nursing effort-associated categories (modified after (Schöning, 1995)).

INPULS-category	nursing time per 24h [min]	nursing time per 24h [h]	description
1	440	7.33	monitoring patient without complications
2	504	8.40	monitoring patient with complications of concomitant disease
3	660	11.00	intensive care patient, short ventilation time
4	831	13.85	intensive care patient, mostly ventilated
5	1,215	20.24	intensive care patient, highly comorbid, mostly ventilated
6	1,300	21.66	intensive care patient, ventilated, highly critical

Table S2. Comparison of patient and tumor characteristics and the histopathological results of open vs. robot-assisted partial nephrectomies (PN) in the propensity score matched analysis.

	**open PN (n=54)**	**robotic PN (n=54)**	**p-value**
**patient characteristics**			
age	65 (23; 90)	64.5 (33; 83)	1.000
gender male	37 (68.5%)	41 (75.9%)	0.503
size [cm]	172 (152; 186)	173.5 (155; 193)	0.885
weight [kg]	85 (51; 145)	85 (54; 130)	0.575
body mass index [kg/m²]	27.3 (20.9; 49.6)	28.7 (18.7; 36.2)	0.784
Charlson comorbidity score (CCI)	9 (2; 18)	8 (3; 20)	1.000
**tumor specifics**			
PADUA score	9 (6; 13)	9 (7; 12)	0.919
low-risk (6, 7)	10 (18.5%)	3 (5.6%)	0.065
mid-risk (8, 9)	19 (35.2%)	32 (59.3%)	**0.021**
high-risk (≥ 10)	25 (46.3%)	19 (35.2%)	0.286
tumor size [cm]	4.5 (0.9; 12)	3.4 (0.7; 9.0)	0.141
**malignancy**	37 (68.5%)	42 (77.8%)	0.359
pT			0.868
pT1a	19 (35.2%)	21 (38.9%)	
pT1b	10 (18.5%)	16 (29.6%)	
pT2a	2 (3.7%)	1 (1.9%)	
pT3	6 (11.1%)	4 (7.4%)	
pN1	1 (1.9%)	1 (1.9%)	1.000
pR1	6 (11.1%)	4 (7.4%)	0.727
**histological subtype**			0.596
clear cell	29 (53.7%)	30 (55.6%)	
chromophobe	3 (5.6%)	2 (3.7%)	
papillary	4 (7.4%)	9 (16.7%)	
other malignant	1 (1.9%)	1 (1.9%)	
angiomyolipoma	2 (3.7%)	2 (3.7%)	
oncocytoma	9 (16.7%)	9 (16.7%)	
cyst	2 (3.7%)	1 (1.9%)	
nephritis	3 (5.6%)	-	
other benign	1 (1.9%)	-	

Table S3. Comparison of the average case-related costs for the administration of erythrocyte concentrates after open vs. robot-assisted partial nephrectomy (PN) after propensity score matching.

	**Open PN (n=54)**	**Robotic PN (n=54)**
mean consumption of erythrocyte concentrates	0.7037 / patient	0.2778 / patient
**case-related costs** (145€ / concentrate)	102.04 €	40.28€

Table S4. Comparison of the average material and consumption costs per partial nephrectomy (PN) after propensity score matching.

	**Open PN (n=54)**	**Robotic PN (n=54)**
**material costs**	124.85€	1,264.55€
**sterilization**	104.02€	164.77€
**maintenance costs**	-	359.77€
**sum**	228.87€	1,789.09€
